# Immediate Diagnosis of Breast Carcinoma on Core Needle Biopsy Using Ex Vivo Fluorescence Confocal Microscopy: Feasibility in a One-Stop Breast Clinic Workflow

**DOI:** 10.3390/life14111384

**Published:** 2024-10-28

**Authors:** Marie-Christine Mathieu, Voichita Suciu, Marie-Laure Tanguy, Neila Ines Ben Romdhane, Salma Moalla, Sana Harguem-Zayani, Remy Barbe, Corinne Balleyguier, Angelica Conversano, Muriel Abbaci

**Affiliations:** 1 Department of Medical Biology and Pathology, Gustave Roussy, Université Paris-Saclay, 94805 Villejuif, France; marie-christine.mathieu@gustaveroussy.fr (M.-C.M.);; 2 Surgery and Pathology Photonic Imaging Group, Gustave Roussy, 94805 Villejuif, France; angelica.conversano@gustaveroussy.fr; 3 Department of Biostatistics and Epidemiology, Gustave Roussy, Université Paris-Saclay, 94805 Villejuif, France; 4 Oncostat U1018, Inserm, Université Paris-Saclay, Labeled Ligue Contre le Cancer, 94805 Villejuif, France; 5 Department of Radiology, Gustave Roussy, Université Paris-Saclay, 94805 Villejuif, France; 6 Department of Breast and Plastic Surgery, Gustave Roussy, Université Paris-Saclay, 94805 Villejuif, France; 7 UMS AMMICa 23/3655, Plateforme Imagerie et Cytométrie, Gustave Roussy, Université Paris-Saclay, 94805 Villejuif, France

**Keywords:** pathology, diagnosis, breast cancer, confocal microscopy, fluorescence, one-stop breast clinic

## Abstract

Background: In the one-stop breast clinic setting, breast cytology traditionally provides immediate diagnosis of carcinoma. Fluorescence confocal microscopy (FCM) is an emerging optical technique enabling ex vivo analysis of breast biopsies in real-time. This study represents the first proof of concept for integrating FCM imaging into the routine workflow of breast core needle biopsies (CNB) at Gustave Roussy’s one-stop breast clinic. Methods: Fifty women with breast masses underwent consecutive enrollment. Biopsies were stained with acridine orange and fast green, followed by imaging using the Vivascope 2500M-G4 (FCM). Interpretation was conducted by two pathologists in real time (PT1) or postoperatively (PT2). Concordance with definitive histology, the duration of the FCM protocol, and its impact on conventional histopathology, immunohistochemistry, and FISH analyses were evaluated. Results: In our study of 50 biopsies, a concordant diagnosis of malignancy was performed using FCM on the malignant cases at definitive histology in 93.5% (29/31 cases) and in 90.3% (28/31 cases) according to PT1 and PT2, respectively. When the FCM suspicious cases were added, FCM identified 100% (31/31 cases) and 96.7% (30/31 cases) of the malignant cases according to PT1 and PT2, respectively. A notable false positive case was identified as a complex sclerosing lesion. The median time for sample preparation (including tissue reception) was 5 min, while the median time for imaging acquisition with interpretation was 3 min for PT1, but 1 min required for interpretation alone by PT2. Histopathological alterations were not more prevalent in FCM-imaged biopsies compared to conventionally treated biopsies. The immunophenotyping and molecular assessment of tissue were preserved after FCM protocol. Conclusions: FCM shows promise as a new histological method for the immediate diagnosis of breast carcinoma on core needle biopsies in a one-stop clinic setting, while also preserving tissue specimens for final histology.

## 1. Introduction

One-stop diagnostic facilities, providing same-day diagnosis and integrated multidisciplinary care, have been conducted and implemented since the 1990s to improve the quality and decrease the delays in achieving a diagnosis for suspected breast cancer [1]. When the clinical, radiological, and diagnostic findings consistently indicate malignancy, the patient is informed that the mass is highly likely to be malignant. This initiates a discussion about the potential treatment plan, including surgery, allowing the patient to begin adjusting to the likely diagnosis. The following week, the final histological results are presented at the multidisciplinary breast meeting, and the confirmed diagnosis is communicated to the patient. A consensus treatment plan is also finalized and can be promptly implemented. Gustave Roussy established a large-scale, multidisciplinary one-stop breast clinic in April 2004. Prompt and accurate diagnostic information is given directly from a pathologist specialized in cytology. This translates into improved management of breast cancer patients, characterized by earlier diagnosis and a reduced number of outpatient appointments. The implementation of immediate diagnosis has proven transformative, significantly reducing turnaround time and alleviating patient anxiety [1]. Touch imprint cytology (TIC) of core needle biopsy (CNB) allows for immediate cytological diagnosis and high quality histological assessment from a single diagnostic procedure [2,3,4]. This approach is used for the evaluation of suspicious breast masses categorized as BI-RADS 4b, 4c, and 5 according to the breast imaging reporting and data system, within the Gustave Roussy one-stop breast clinic [5]. 

Fifteen years ago, benchtop confocal microscopy revolutionized the imaging of breast cancer by providing high-resolution images of cells and tissue architecture [6]. Although capable of fluorescence imaging, the technology at the time was hindered by lengthy acquisition times and a limited field of view, which precluded its clinical application. However, recent advancements in ex vivo fluorescence confocal microscopy (FCM) have overcome these challenges. FCM now offers the rapid acquisition of high-resolution images from fresh, thick tissue specimens, facilitated by optimized ergonomics and user-friendly software [7]. In a recent study focused on breast cancer imaging, pathologists demonstrated high sensitivity and specificity in interpreting FCM images obtained from surgical specimens using dedicated large-sample imager [8]. This technology is particularly valuable for assessing tumor margins in breast lumpectomy procedures [9,10]. However, there is also significant potential in providing rapid histological interpretations of fresh samples shortly after patient examination, following a simple staining process. Elfgen et al. demonstrated high accuracy in interpreting breast carcinoma in core needle biopsies [11]. In oncology, the ability to obtain a reliable histopathologic diagnosis quickly could offer a substantial clinical advantage in the management of patients undergoing one-day breast diagnosis [12]. The recent literature on FCM for imaging breast surgical specimens further underscores the relevance of evaluating this technology for use in one-stop breast clinics [13].

We conducted the first proof-of-concept study to assess the feasibility of FCM imaging on breast CNB within the routine workflow of a one-stop breast clinic. Our study aimed to achieve several critical objectives. First, we sought to evaluate the concordance rate between FCM imaging diagnoses and definitive histology. This evaluation involved a blinded review of FCM images of breast biopsies by two pathologists. Second, we conducted a time analysis to assess how FCM could be integrated into the routine workflow of the one-stop breast clinic at Gustave Roussy. Lastly, we focused on evaluating the quality of samples post-imaging to determine their suitability for downstream histological examinations.

## 2. Materials and Methods

### 2.1. Study Population

This prospective ex vivo study was conducted at Gustave Roussy’s one-stop breast clinic in Villejuif, France. Approval was obtained from the Gustave Roussy breast committee and the institutional review board for the present work (reference 2023-276). Between October 2023 and November 2023, fifty women with masses categorized as BI-RADS 3, 4a, 4b, 4c, or 5 were consecutively enrolled according to the Breast Imaging-Reporting and Data System of the American College of Radiology [14], as follows: BI-RADS 0—insufficient or incomplete study; BI-RADS 1—normal study; BI-RADS 2—benign features; BI-RADS 3—probably benign (<2% risk of malignancy); BI-RADS 4—suspicious features (divided into categories 4a, 4b, and 4c depending on the likelihood of malignancy); BI-RADS 5—probably malignant (>95% chance of malignancy); and BI-RADS 6—malignant (proven malignant on tissue biopsy).

There was no selection based on BI-RADS classification or the size of the mass. In accordance with French Good Clinical Practices guidelines for non-interventional studies involving ex vivo human tissue, patients were provided with an information sheet during their medical visit, and this was subsequently added to their records.

### 2.2. Patient Evaluation

On the same day of their visit to the one-stop breast clinic, patients underwent ultrasound-guided biopsy following radiologic examination. Typically, three consecutive core biopsies (either 14G or 10G) were performed on the identified mass. Biopsy thickness varied between 1.6 mm and 2.5 mm based on needle gauge. All biopsies were 10–20 mm in length. Among these biopsies, two underwent touch imprint cytology for immediate diagnosis. Additionally, one of these two biopsies was analyzed using FCM.

Subsequently, all biopsies were formalin-fixed and paraffin-embedded (FFPE), and stained with hematoxylin/eosin/saffron (HES) for definitive histology according to established guidelines. To accurately assess the time required for FCM analysis of a single nodule within the clinic’s workflow, patients with biopsies involving multiple breast nodules were excluded from this specific time measurement.

### 2.3. Confocal Microscopy

Biopsies were imaged using the Vivascope 2500M-G4 (Vivascope GmbH, Munich, Germany), a clinical-grade confocal scanning microscope. This device features two laser diodes operating at 488 nm and 638 nm for fluorophore excitation, combined with fluorescence signal collection. The microscope is equipped with a x38 objective water immersion with a numerical aperture of 0.85 (Caliber I.D. StableView). It achieves a maximum magnification of ×550, producing reconstructed images from a mosaic of individual scans, with a maximum total scan area of 34 × 25 mm. Each single field of view offers a resolution of 1024 × 1024 pixels, corresponding to a pixel size of 0.5 µm. The Vivascope 2500M-G4 system allows for z-plane imaging from 0 to 200 µm, utilizing fluorescence signal collection with an axial resolution of 4 µm. In addition to confocal imaging, the system features a camera that captures a white-light macro image of the sample. Integration of the confocal and macroscopic images is facilitated through precise correlation, aided by a pointer that enables seamless navigation between the two views. The final fluorescence images are presented in a format akin to H&E staining, where nuclei are depicted in purple, while cytoplasm and extracellular structures appear pink. 

### 2.4. Tissue Processing and Staining 

The fresh biopsy was carefully placed on a foam support and rinsed with 0.9% NaCl solution. Subsequently, the staining process involved topical application in the following sequence, repeated twice with brief intervals of few seconds between each step: rinsing with 70% ethanol, applying 5 drops of acridine orange at 0.025% (the solution was prepared by diluting a 1% stock solution (Morphisto, Offenbach, Germany) with 0.9% NaCl), followed by 5 drops of fast green at 0.067% (the solution was prepared by diluting a 0.1% stock solution (Morphisto, Offenbach, Germany) with 0.9% NaCl), and final rinsing with 0.9% NaCl solution. 

Acridine orange is a cell-permeating nucleic acid-binding dye that emits green fluorescence when bound to DNA (λ excitation = 502 nm and λ emission = 525 nm). Previous studies have successfully described acridine orange dye for ex vivo microscopy for histologic diagnosis [10,15]. Fast green is highly selective for collagen (λ excitation = 619 nm and near-infrared fluorescence emission when bound to proteins) and was reported in previous fluorescence microscopy examination [16].

Next, the foam carrying the tissue sample was sandwiched between two glass slides, ensuring gentle and complete contact between the tissue, foam, and glass slides. Blu-tack was used to securely connect the glass slides in a “sandwich” configuration. This assembly, with the tissue in contact with the glass slide on the objective side, was then mounted onto the microscope sample holder for en face image acquisition.

Following image acquisition, the core biopsy was promptly fixed in 10% buffered formalin. Subsequently, each imaged biopsy was embedded individually in a paraffin block. Formalin-fixed paraffin-embedded tissue processing preserves tissue for long-term analysis. It involves several steps, fixation, dehydration, and inclusion in paraffin. Breast biopsies were typically fixed in formalin from 6 to 18 h. Sections were cut at two levels (3 µm thick, 60 µm apart) and stained with hematoxylin/eosin/saffron (HES) for definitive histology using standard methodologies. Other sections could be added for immunohistochemistry to refine the diagnosis and to evaluate biomarkers in invasive carcinomas.

The definitive histological diagnosis was performed on HES sections according to the WHO classification of breast tumors [17]. Immunohistochemistry was used to refine the diagnosis and to evaluate biomarkers in invasive carcinomas [18].

### 2.5. Image Review and Interpretation

Two pathologists conducted histomorphologic interpretation of the 50 FCM images in a blinded manner. Both pathologists were unaware of the touch imprint cytology diagnosis and the definitive histological diagnosis during their assessments.

**PT1 (MCM)**: A senior pathologist, specializing in breast cancer and experienced in optical imaging [8,19], reviewed the FCM images immediately after acquisition within the clinical workflow of the one-stop breast clinic. 

**PT2 (VS)**: Another senior pathologist specializing in breast cancer, without prior experience in optical imaging, reviewed the images remotely, four weeks after the examination. 

Neither pathologist had received specific training in interpreting FCM images of breast biopsies. They were provided with clinical and imaging data, including BI-RADS classification and patient records (age, medical history, mass localization, and size). The interpretation followed conventional histopathological criteria to establish diagnoses based on the FCM images.

Each pathologist completed the following interpretation sheet for the FCM images:

- The acquisition mode of the specimen was reported as either “Vivablock” for a single image at one depth, or “Vivacube” for a z-series comprising one image at three depths spaced 12 µm apart.
- The quality of the FCM image was assessed using the following categories: limited quality, moderate, good, and very good, facilitating confident sample interpretation.
- FCM image B-Classification according to the reporting system of Ellis for minimal invasive biopsies, as follows: B1: normal tissue; B2: benign; B3: benign but of uncertain malignant potential; B4: suspicious of malignancy; and B5: malignant [3].

Histopathological diagnosis on FCM images according to:

- **Normal**
- **Benign**: mastopathy, fibroadenoma, papilloma, inflammation, other benign conditions;
- **Atypical**: atypical ductal hyperplasia, atypical lobular hyperplasia, other atypical conditions;
- **Malignant**: ductal carcinoma in situ (DCIS), lobular carcinoma in situ (LCIS), invasive carcinoma of no special type (IC-NST), invasive lobular carcinoma (ILC), other malignant conditions.

The B classification and histopathological diagnosis on FCM images were compared directly with the definitive B classification and histological diagnosis performed on the corresponding HES slide, which served as the gold standard. Concordance between the diagnoses was based on whether the B-category (benign or malignant) assigned by FCM imaging matched that of the definitive histology. Minor discrepancies were noted when the B-category (benign or malignant) was the same in both FCM and histology, but the specific type of lesion differed. Major discrepancies were recorded when the B-category assigned by FCM imaging differed from the B-category in the definitive histological diagnosis (benign versus malignant).

### 2.6. Histology and Immunohistochemistry

The quality of the histopathological sections following fluorescence staining and “sandwich mounting” for FCM image acquisition was assessed.

Immunohistochemical (IHC) evaluation of biomarkers (estrogen receptor (ER), progesterone receptor (PR), HER2, and Ki67) was performed in invasive carcinomas and used for patient selection for chemotherapy, hormonal treatment, and anti-HER2. The staining was conducted on a Ventana platform (Ventana BenchMark Ultra system, Tucson, AZ, USA) following the manufacturer’s instructions. The antibodies used included estrogen receptor (ER) (clone SP1, Ventana), progesterone receptor (PR) (clone 1E2, Ventana), HER2 (clone 4B5, Ventana), and Ki67 (clone MIB1, Dako), applied to all biopsies performed, including those analyzed via FCM.

Tumors were categorized as ER- or PR-positive based on French guidelines, which define positivity as at least 10% of invasive tumor cells displaying clear nuclear staining, regardless of staining intensity. HER2 expression was assessed using ASCO guidelines [20], with scores ranging from 0 to 3+. Ki67 positivity was determined by the presence of at least 20% invasive tumor cells displaying clear nuclear staining, independent of staining intensity. 

For tumors scoring 2+ for HER2, fluorescence in situ hybridization (FISH) was performed using the Her2/Cep17-G probe kit (Vysis). A positive result was determined if HER2 gene amplification was detected by FISH.

The corresponding HES, IHC, and Ki67 slides were digitized using a digital pathology system, specifically scanned viaa NanoZoomer S210 Scanner (Hamamatsu Photonics, Massy, France). 

## 3. Results

### 3.1. Clinical and Histopathological Data

Fifty patients were enrolled in the protocol. The BI-RADS classification of the 50 breast masses that underwent biopsies was as follows: BI-RADS 5—19 cases, BI-RADS 4—28 cases (6 BI-RADS 4a, 11 BI-RADS 4b, 11 BI-RADS 4c), and BI-RADS 3—3 cases. The final histological diagnosis revealed carcinoma in 35 patients (70%), including 2 cases of DCIS (ductal carcinoma in situ), 24 cases of IC-NST (invasive carcinoma of no special type), 6 cases of ILC (invasive lobular carcinoma), 1 case of micropapillary carcinoma, 1 case of metastasis from renal carcinoma, and 1 case of papillary carcinoma. Benign lesions were diagnosed in the remaining 15 patients (30%), comprising 2 cases of mastopathy, 4 cases of fibroadenomas, 2 cases of inflammation, 1 case of ductal hyperplasia without atypia, 1 case of cylindric metaplasia, 2 cases of adenosis, 1 case of a cyst, 1 case of gynecomastia, and 1 case of complex sclerosing lesion. 

In four cases, carcinoma was not detected in the core biopsy imaged by FCM, but was found in another tissue fragment biopsy. Therefore, only 31 malignant cases were imaged by FCM in total.

### 3.2. FCM Image Interpretation

**Figure 1:**
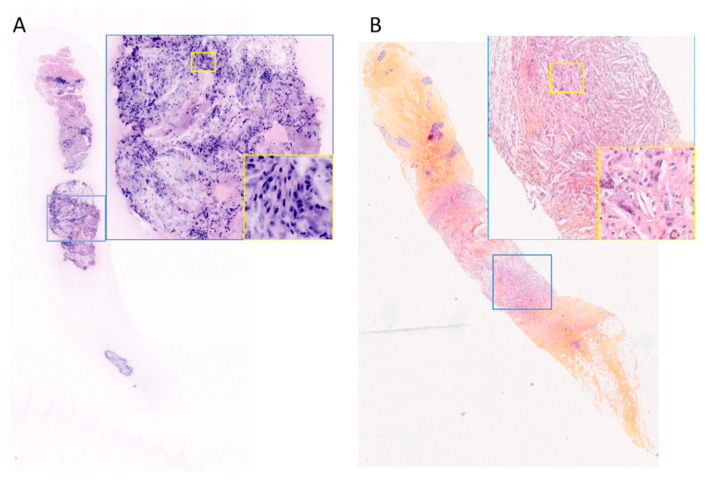
(**A**). Fresh tissue—FCM image not interpreted by PT2. (**B**). FFPE tissue—corresponding HES slide interpreted as inflammation by PT1, magnification ×15—blue inset magnification ×100 and yellow inset magnification ×400.

All 50 fresh biopsies underwent FCM imaging, resulting in a single image per sample presented to the pathologists. These 50 images were independently analyzed by PT1 and PT2. One image was deemed insufficiently informative by PT2 and therefore was not interpreted, primarily due to poor contact of the sample in the lower part (Figure 1A). The quality of the obtained images was considered good or very good in 41 cases (82%) for PT1 and 36 cases (72%) for PT2. In nine cases (18%) reviewed by both pathologists, the image quality was moderate. For PT2, the image quality was limited in five cases (10%), including the case that was not interpreted. Limited or moderate quality was attributed to fluorescence dye staining that was either too intense or too light, and horizontal lines observed on the FCM images due to ongoing construction work in the hospital, which introduced acquisition artifacts.

The correlation between the B-classification of FCM images and corresponding H&E-stained (HES) sections is summarized in Table 1.

Among the 31 malignant cases (B5) at definitive histology, malignancy (B5) by FCM was diagnosed in 93.5% (29/31 cases) and in 90.3% (28/31 cases) according to PT1 and PT2, respectively (Table 1).

**Figure 2:**
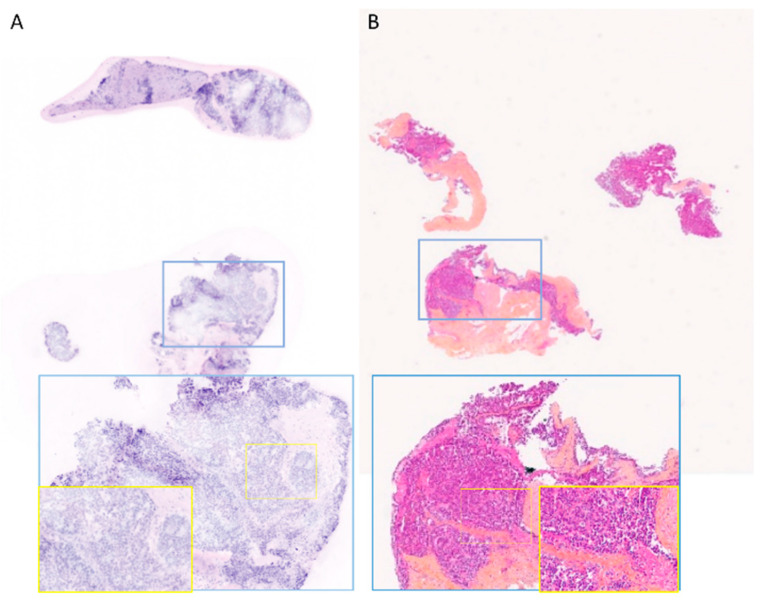

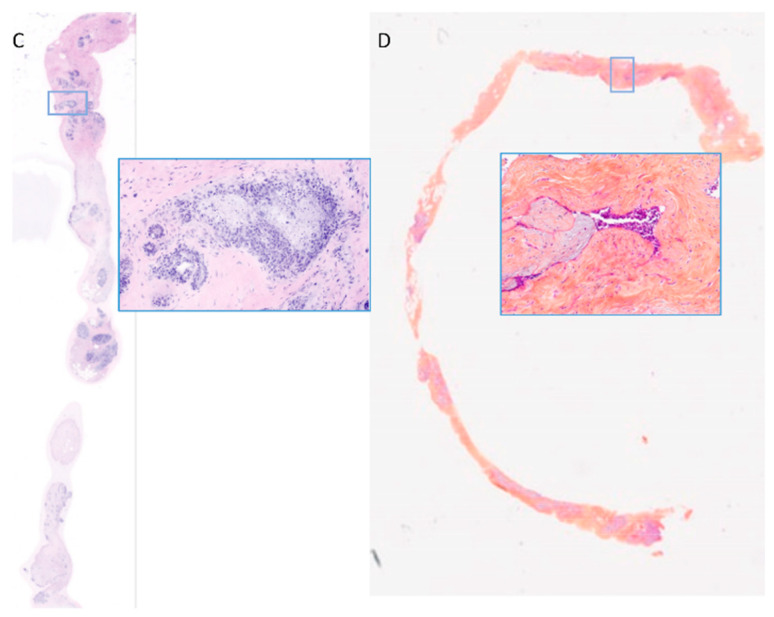
FCM images interpreted as suspicious (B4) by PT1 or PT2 (**A**). Fresh-tissue—papillary carcinoma. (**B**). FFPE tissue—corresponding histological section (**C**). Fresh tissue—Fibroadenoma (**D**). FFPE tissue—corresponding HES section. Magnification ×15—blue inset magnification ×100—yellow inset magnification ×200.

Initially, six biopsies were classified as suspicious (B4) by PT1 and two by PT2. Following confirmation that these suspicious B4 cases were indeed malignant (2/6 for PT1 and 2/2 for PT2), the overall diagnosis of malignancy improved to 100% (31/31 cases) for PT1 and 96.7% (30/31 cases) for PT2 (Figure 2A). PT2 did not identify one malignant biopsy out of thirty-one on FCM images. 

For the remaining four B4 cases reviewed by PT1, a re-evaluation of FCM images with the assistance of corresponding HES sections and patient reports was conducted. Among these cases, PT1 noted foci of sclerosing adenosis that were challenging to differentiate from well-differentiated invasive carcinoma, and three cases of fibroadenoma were identified as B4. However, in one benign case, PT1 observed granular material in the duct suggestive of tumor necrosis, while PT2 interpreted an intraductal proliferation as atypical ductal hyperplasia (Figure 2C).

The diagnostic agreement between the two pathologists regarding the FCM images for categories B1+B2+B3 versus B5 (benign versus malignant lesions) at both the one-stop and consolidated diagnoses was 97.5% (40/41 cases). 

**Figure 3:**
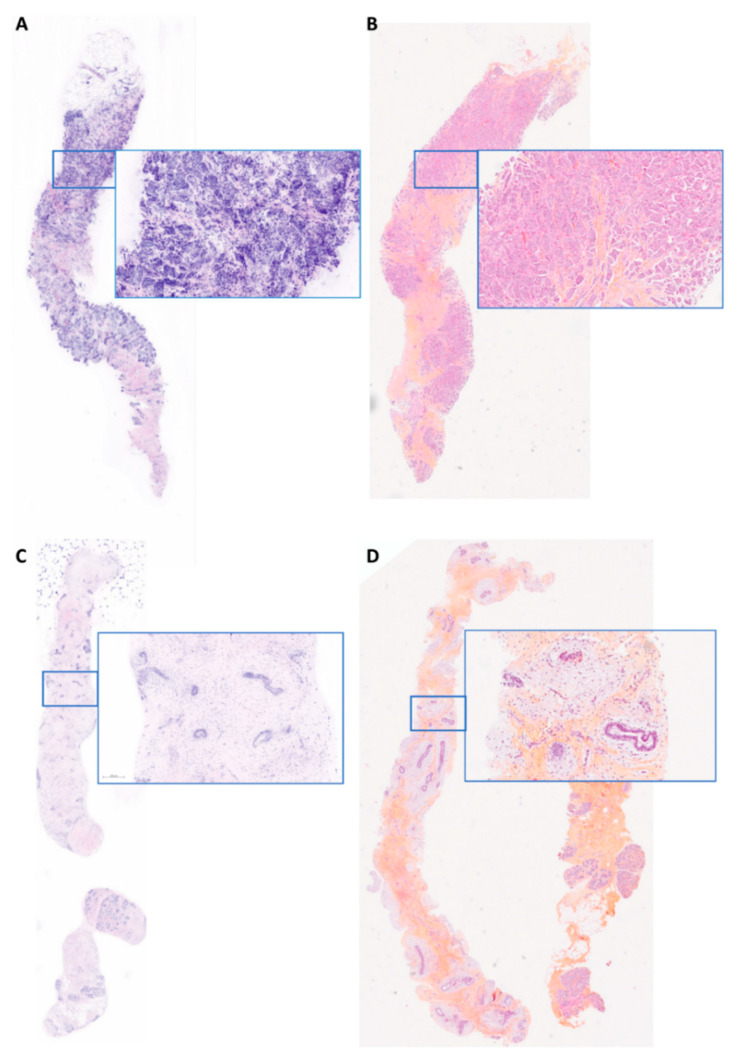
Examples of FCM images correctly interpreted by PT1 and PT2 (**A**). Fresh tissue—IC-NST. (**B**) FFPE tissue—corresponding HES slide diagnosed as IC—micro papillary subtype. (**C**). Fresh tissue—fibroadenoma (**D**). FFPE tissue—corresponding HES slide. Magnification ×15—blue inset magnification ×100.

The correlation between the histological diagnosis on FCM images and the corresponding H&E-stained (HES) sections, considered the gold standard, is detailed in Table 2. Both pathologists (Figure 3) correctly diagnosed all 29 invasive carcinomas. Regarding histological subtypes, PT1 and PT2 correctly identified all 21 cases of invasive carcinoma of no special type (IC-NST). Among the lobular carcinomas, PT1 identified four out of six cases correctly, while PT2 identified three out of six cases correctly as lobular carcinoma. For special types, both pathologists classified a micropapillary carcinoma as IC-NST (Figure 2A). However, PT2 correctly identified a metastasis from renal carcinoma, whereas PT1 classified it as IC-NST or apocrine carcinoma.

**Figure 4:**
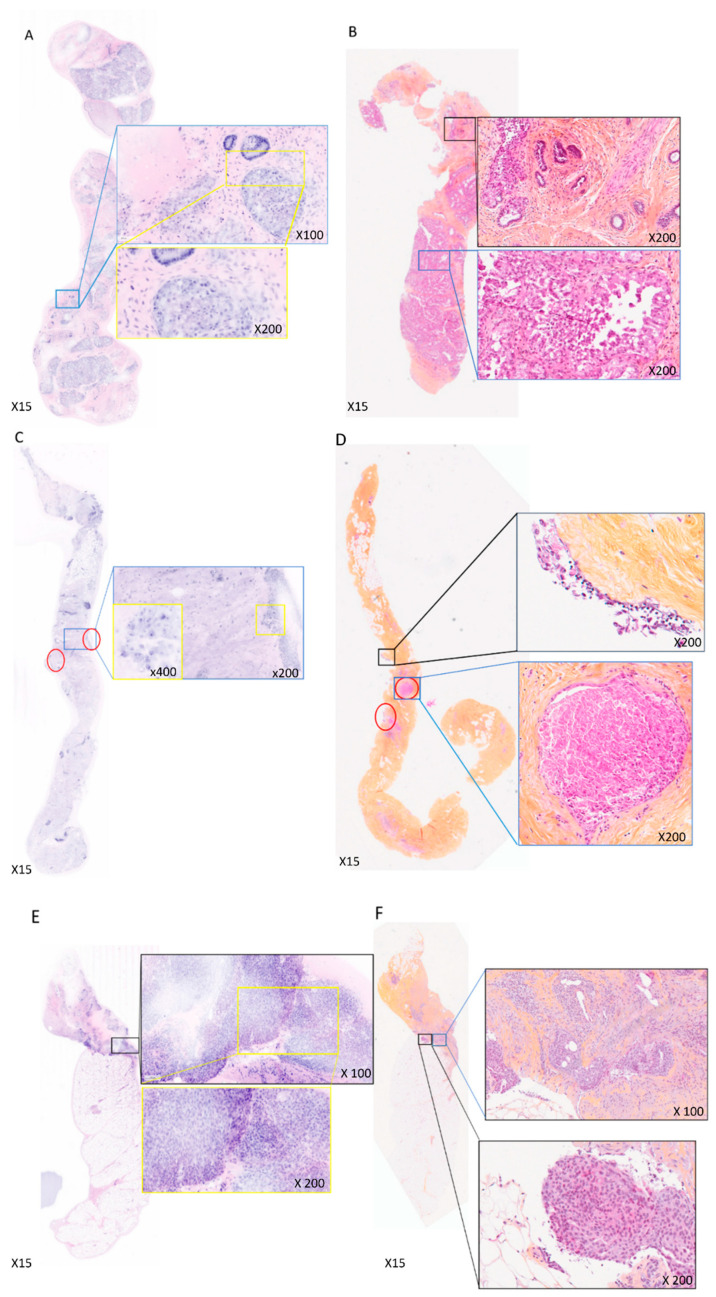
FCM images interpretation showed major discrepancies with the final diagnosis. (**A**). Fresh tissue—fibroadenoma with lactating metaplasia interpreted as DCIS by PT1. (**B**). FFPE tissue—corresponding HES section. (**C**). Fresh tissue—DCIS interpreted as suspicious by PT1 and as mastopathy by PT2. In red circles: clusters of suspicious cells observed by PT1. (**D**). FFPE tissue—corresponding HES section with the two ducts leading to the final diagnosis of DCIS circled in red. (**E**). Fresh tissue—complex sclerosing lesion interpreted as DCIS invasion by PT1 and as DCIS by PT2. (**F**). FFPE tissue—corresponding HES section.

During a re-evaluation of the FCM images with the support of corresponding H&E-stained (HES) sections and patient reports, false negatives and false positives were analyzed (Table 3). PT2 encountered one false negative case on FCM, which was identified as DCIS (ductal carcinoma in situ) on definitive histology (Figure 4C), where only two ducts showed evidence of DCIS (Figure 4D). Upon re-examination of the FCM image, these ducts were not visible in the z-plan confocal image, leading to the false negative diagnosis for PT2. For PT1, aggregates of cells with large and irregular nuclei were observed surrounding the core biopsy, leading to a suspicious of malignancy (B4) classification (Figure 4C). The false positives included two benign lesions. PT1 diagnosed a fibroadenoma with significant lactating metaplasia as DCIS (Figure 4A,B), whereas PT2 recognized it correctly as lactating metaplasia. In another case (Figure 4E), a complex sclerosing lesion with florid epithelial hyperplasia was diagnosed as in situ carcinoma with invasion by PT1 and as DCIS by PT2 (Figure 4E,F). 

### 3.3. FCM Protocol Duration 

The duration of the FCM protocol from biopsy reception (including administrative tasks) to tissue preparation (fluorescence staining and “sandwich mounting”) ranged from 2 to 17 min, with a median time of 5 min and a mean time of 5 min and 43 s (95% confidence interval (CI) [4 min and 44 s; 6 min and 43 s]). In two exceptional cases, a delay of 17 min occurred when biopsies from two patients arrived simultaneously for analysis. The second sample was processed only after the first one had been interpreted and fixed in formalin following image acquisition.

The process of image acquisition typically required one to two acquisitions, primarily to select the appropriate z-plane for obtaining a satisfactory image. The time for image acquisition ranged from 1 to 13 min, with a median of 3 min and a mean of 3 min and 43 s (95% CI [3 min and 7 s; 4 min and 30 s]) for PT1. For image interpretation by PT2, the time ranged from 1 to 6 min, with a median of 1 min and a mean of 1 min and 36 s (95% CI [1 min and 16 s;1 min and 55 s]). A delay of 6 min occurs for a FCM image case which was classified B4. Diagnosis based on FCM images was made following the first image acquisition in 38 cases (76%), after the second image acquisition in 9 cases (18%), after one image and a z-series in 1 case (2%), and after two images and one z-series analysis in 2 cases (4%), aimed at obtaining the most representative image of the sample.

### 3.4. Assessment of Biopsy Quality, Immunohistochemical Staining, and FISH Evaluation Following FCM Imaging

The histopathological analysis remained uncompromised by the FCM protocol (staining and imaging steps), as no significant changes were observed in the H&E-stained (HES) sections. Specifically, 37 FCM biopsies showed no alteration compared to 36 directly fixed biopsies. Light alterations, such as hyperchromatism of nuclei, cell crushing, and cell retraction, were reported in the remaining samples (13 with FCM protocol and 14 without) (Table 4). Importantly, no specific alterations attributable to the imaging procedure were identified.

Concordance in the classification of ER and PR positivity or negativity was maintained between specimens imaged with FCM and those directly fixed in formalin. 

For HER2, the scoring was consistent between specimens imaged with FCM and those directly fixed in formalin in nine out of twelve cases. One tumor was classified as score 0 on the imaged specimen and 1+ on the non-imaged specimen. Conversely, two specimens were classified as score 2+ on the imaged specimen and 1+ on the non-imaged specimen. In these two cases, membranous staining was complete and moderate in the imaged specimens, whereas it was incomplete and ranged from low to moderate in the non-imaged specimens.

Ki67 staining showed concordance in nine out of twelve cases. In the discordant cases, Ki67 expression percentages were 10%, 20%, and 22% in the imaged specimens compared to 20%, 15%, and 15% in the non-imaged specimens, respectively. The maximum difference in Ki67-positive cell percentages between imaged and non-imaged specimens was 15%, with a median difference of 0% (Table 5).

FISH evaluation was conducted in three cases, with similar results observed between specimens imaged with FCM and those directly fixed in formalin. In all cases, HER2 gene amplification was not detected. The HER2/CTR17 ratio, used for determining FISH positivity, was concordant between the imaged and non-imaged specimens (Table 6).

## 4. Discussion

### 4.1. FCM Data Analysis Concordance Rate between FCM Imaging Diagnoses and Definitive Histology

This study represents the first demonstration of the feasibility of using FCM for the histological analysis of core biopsies from suspicious breast masses in a one-stop clinic setting. The interpretation process was straightforward and intuitive, requiring no prior training, as was the case for PT2. Nackenhorst’s study indicated that the resolution of FCM images obtained with a Vivascope is comparable to that of frozen sections. However, FCM images typically provide less detailed visualization of cytoplasmic structures and the extracellular matrix compared to final histology [13]. Nevertheless, we achieved a high rate in detecting malignancy with FCM. When FCM suspicious and malignant cases are added, FCM identifies 100% (31/31 definitive malignant cases) and 96.7% (30/31 cases) according to PT1 and PT2, respectively. These results compare favorably to those reported by Elfgen, who achieved a malignancy detection concordance rate of 91% (63/69 cases) on CNB [11]. It is important to note that Elfgen’s study included a smaller number of cases (n = 24) [11]. 

While 30.9% of FCM images exhibited limited to moderate quality, image interpretation was not hindered significantly. Only one case could not be interpreted by PT2. Independently of the Vivascope system, ongoing construction near Gustave Roussy hospital caused significant microvibrations during FCM image acquisition. These vibrations affected FCM acquisitions by introducing repeated horizontal lines into the images, which were identified as artifacts in the final FCM images. Additionally, in some cases, fluorescent staining appeared either too intense, heterogeneous, or inadequate due to incomplete contact of the biopsy with the imaging window. A learning curve was observed for tissue preparation, particularly for the sandwich mounting technique and image acquisition, aimed at optimizing FCM image quality. Importantly, an antivibrational table has been integrated into the Vivascope system to mitigate horizontal line artifacts in future studies.

PT1 and PT2 failed to identify the sole case of DCIS on the FCM image, as the two invaded ducts identified on the corresponding HES section were absent in the confocal image. It is noteworthy that aggregates of tumor cells around the core biopsy were diagnosed as suspicious of malignancy by PT1. Although the promising global results from PT2 did not highlight the importance of a learning curve, the experienced PT1 suspected malignancy in the DCIS case due to the presence of detached, suspicious cells, which are frequent in biopsy tissues containing DCIS with necrosis. The two false positive cases were not attributable to FCM imaging but rather represented challenging diagnostic cases well known by pathologists. A common FCM false positive was a complex sclerosing lesion, where the final histopathological diagnosis had required the immunohistochemical identification of myoepithelial cells to confirm the benignity. Furthermore, PT1’s second false positive was a fibroadenoma with significant lactating metaplasia, a rare lesion characterized by large nuclei, prominent nucleoli, and anisonucleosis, contributing to the misinterpretation. In this case, the young age of the patient provided a crucial clue for guiding PT2 towards a diagnosis of benignity. Additionally, for false positive cases, reviewing medical files and experience of FCM images particularly in challenging cases will be helpful for improving the diagnosis.

### 4.2. Time Analysis and Samples Processing

Our study demonstrated rapid breast biopsy analysis, with processing, acquisition, and interpretation completed in under 8 min by a pathologist. This swift turnaround time aligned with the goals of a one-stop breast clinic, enabling immediate diagnosis. The FCM installation in the one-stop breast clinic and its evaluation in “real conditions” for data acquisition and image interpretation streamline the intense workflow. Moreover, our feasibility study shows that FCM could fit into the existing clinical workflow providing a low occurrence of unsatisfactory samples (1/50 for PT2) or false negative cases leading adaption capabilities. Research on other cancers has explored the potential of FCM for rapid, intraday diagnosis; Razi et al. emphasized the high diagnostic quality of FCM in skin cancer imaging [21]. Amandoeira et al. reported on FCM’s application in imaging solid pancreatic lesions via endoscopic ultrasound-guided fine-needle biopsy, achieving 83.8% adequacy compared to final histology [22]. Titze et al. demonstrated that combining FCM with MRI-guided targeted prostate biopsy enables rapid preliminary diagnosis and enhances clinical management, with intraday diagnosis of clinically significant prostate carcinomas achieved in 68% of cases [23]. The short learning curve for FCM was evident, as pathologists became proficient in image acquisition and data interpretation after just half a day of training. This suggests that integrating FCM into routine practice would not require extensive staff training. 

Compared to touch imprint cytology, FCM could offer most of the time a complete histological diagnosis, with final histology serving as a confirmatory step. FCM provides additional architectural information, potentially enabling more precise identification of carcinoma type (in situ vs. invasive). This technology, offering “multiple virtual sections” through depth imaging in a sample, provides a distinct advantage over imprint cytology. FCM analysis was predominantly conducted on a single optical plane, but multiple optical planes were utilized when necessary. This capability allowed for image acquisition at different depths within the tissue, providing pathologists with the option to interpret images with the highest available information, sometimes from deeper layers of the tissue. In this study, multiple optical planes were employed 12 times to interpret lesions. Consequently, the representativeness of lesions in biopsy images could be enhanced by FCM without requiring additional tissue processing. 

Finally, FCM did not modify the quality of the specimen for definitive histology, ER, PR, HER2, Ki-67 immunohistochemical staining, and HER2 FISH evaluation in our study. FCM does not impose any restrictions on subsequent conventional histology, immunohistological staining, or FISH examinations. Importantly, the entire sample analyzed by FCM remained available for subsequent analyses, which is crucial for managing small tissue samples. These findings align with previous studies by Verri et al. and Titze et al., which also assessed the quality of tissue specimens following FCM imaging [24,25]

### 4.3. FCM for One-Stop Breast Clinic

Recent studies have highlighted the utility of FCM for ex vivo analysis, demonstrating high sensitivity and specificity in the pathology management of diverse conditions. Histopathological diagnosis is based on the identification of morphological criteria validated within the pathologists’ community. Therefore, the recognition of these same criteria in fluorescence imaging by FCM is a guarantee of rapid adoption of the technology. We first describe the application of FCM in one-stop breast clinic with an integrated multidisciplinary care. FCM offers a powerful tool for the diagnosis of breast cancer due to its ability to provide high-resolution and at multiple depths images of biopsy. The high-resolution images enable detailed visualization of cellular structures and features that may be challenging to discern with imprint cytology. Currently, breast mass diagnosis via touch imprint cytology (TIC) of core needle biopsy (CNB) demonstrates high sensitivity (92–97%) and specificity (89–96%) rates [2,3,4]. Inadequate cellularity is observed in 5 to 11.4% of cases with TIC [2,3,4]. Based on our first results, FCM technology has the potential to serve as an alternative to cytological diagnosis, particularly in pathology settings lacking cytopathologists, by providing optical histological sections from breast biopsies within minutes. Failed staining was not observed in our study and was not reported in the literature [8,13,26]. However, in the event of a failed fluorescence staining, biopsies would not be taken again from the patient. The protocol would then be the same as for touch imprint cytology currently, the biopsy analysis would be considered inadequate, and the report would state ‘awaiting definitive histology’. 

FCM enables the classification of carcinomas into in situ and invasive types, as well as the identification of various subtypes of invasive carcinomas. This information is valuable for the patient management. The rapid diagnosis allows a first orientation of the patient to a standard care pathway and decrease the delay of patient care. Usually, if two pathologists disagree on a diagnosis of an FCM image, a third pathologist should be consulted. However, in a one-stop breast clinic, time constraints may need to wait definitive histology. Nonetheless, the rapid FCM diagnosis will always be confirmed by definitive histology and complemented by immunohistochemical or FISH evaluation. With FCM system featuring two excitation wavelengths, future developments may incorporate fluorescence morphological imaging combined with immunofluorescence imaging in each emission channel. This integration could offer complementary information.

A very promising aspect of FCM is its potential role in extemporaneous telepathology, which is becoming increasingly relevant as pathology departments worldwide consolidate into fewer, more specialized diagnostic centers [13]. Given the anticipated shortage of pathologists in the future, this approach could be crucial. Tissues could be processed and imaged at radiology centers without on-site pathology departments and then analyzed in real-time by specialized pathology centers remotely. Our study suggests that FCM represents a significant advancement in the imaging arsenal of one-stop breast clinics, enabling patients to receive histological results on the same day.

A limitation of our study is that we did not assess the cost-effectiveness of FCM. We plan to address this in future research. While FCM represents a substantial investment, a single device can be shared for various one-day diagnostic applications, such as breast, thyroid, prostate, and pancreatic cancers [22,25,27]. A biopsy sample imaged by FCM is preserved in its entirety for subsequent conventional histological and biological analysis. By providing real-time information, FCM could eliminate the need for multiple biopsies to acquire cancer tissue, sparing patients from the discomfort and risks associated with multiple biopsies. In a therapeutic context, real-time imaging of surgical margins during breast-conserving surgery using FCM is being investigated in numerous trials to identify and remove any residual cancer cells [9,10,28,29]. This would reduce the need for multiple surgeries and increase the likelihood of complete resection. Despite the cost, the return on investment could be significant, particularly if FCM is integrated into a diagnostic and therapeutic approach to enhance the perspective of healthcare provider.

## 5. Conclusions

Based on the study results, our work demonstrates the feasibility of immediate histological diagnosis of core biopsies of suspect breast masses using FCM for the first time in a one-stop clinic. FCM provides rapid histological interpretation without the need for tissue section preparation, delivered within minutesafter patient examination. Our findings underscore the potential to integrate FCM procedures for patients requiring CNB, offering immediate histological interpretations within the routine workflow of a one-stop breast clinic. 

To further assess whether FCM analysis can facilitate clinicians in delivering definitive diagnoses to patients within a single day, our next study will focus on measuring the diagnostic quality parameters of FCM in a larger patient cohort within a one-stop breast clinic setting, including considerations of financial sustainability.

## Figures and Tables

**Table 1:** Correlation between B classification on FCM images and corresponding HES slides.

		Pathologist 1 B-Classification on FCM Images					Pathologist 2 B-Classification on FCM Images						Total
		B1	B2	B3	B4	B5	B1	B2	B3	B4	B5	NI	
**B-classification on definitive** **histology**	B1	3	-	-	-	-	2	1	-	-	-	-	3
	B2	-	10	-	4	1	-	11	3	-	-	1	15
	B3	-	-	-	-	1	-	-	-	-	1	-	1
	B4	-	-	-	-	-	-	-	-	-	-	-	-
	B5	-	-	-	2	29	-	1	-	2	28	-	31
	Total	3	10	0	6	31	2	13	3	2	29	1	50

With B1: normal, B2: benign lesion, B3: uncertain malignant potential, B4: suspicious lesions, and B5 malignant lesions. NI: biopsy not interpreted.

**Table 2:** Correlation between histological diagnosis on FCM images and corresponding HES slides.

		Pathologist 1 FCM Image Interpretation					Pathologist 2 FCM Image Interpretation						Total
		Normal	Benign	Atypical/Suspicious	DCIS	IC	Normal	Benign	Atypical/Suspicious	DCIS	IC	NI	
Histological type on definitive histology *	**normal**	2	-	-	-	-	2	-	-	-	-	-	2
	benign	1	11	3	1	1		14	1	1	-	1	17
	atypical/ suspicious	-	-	-	-	-	-	-	-	-	-	-	-
	DCIS	-	-	1	-	-	-	1	-	-	-	-	1
	IC	-	-	-	-	29	-	-	-	-	29	-	29
	Total	3	11	4	1	30	2	15	1	1	29	1	49 *

* One case of papillary carcinoma could not be classified as in situ or invasive on biopsy at definitive histology. It was diagnosed as papillary carcinoma by PT1 and DCIS by PT2. NI: biopsy not interpreted.

**Table 3:** True negative, true positive, false positive, and false negative after suspicious cases exclusion (B1 + B2 + B3 versus B5).

	Pathologist 1 (n = 44)	Pathologist 2 (n = 47)
**True negative**	100% (13/13)	94.4% (17/18)
**True positive**	93.5% (29/31)	96.5% (28/29)
**False positive**	6.5% (2/31)	3.5% (1/29)
**False negative**	0 (0/13)	5.6% (1/18)

**Table 4:** Alterations identified in the HES sections.

Alteration	FCM Biopsy	Directly Fixed Biopsy
No alteration	37	36
Nuclear hyperchromatism	8	10
Cells crushed	4	4
Cell retraction	1	-
**Total**	50	50

**Table 5:** Comparison of biomarkers expression between specimens imaged with FCM and directly fixed.

Biomarker	Specimen Imaged	Specimen Not Imaged
**ER**		
negative	3	3
positive	9	9
**PR**		
negative	2	2
positive	10	10
**HER2**		
score 0	1	2
score 1+	6	3
score 2+	3	5
score 3+	2	2
**Ki67**		
≤5	1	-
10–25	5	5
≥25	6	7
<20	3	2
≥20	9	10

**Table 6:** Comparison of HER2 ratio between specimens imaged with FCM and directly fixed.

Number of the Case	Ratio HER2/CTR17 in the FCM Sample	Ratio HER2/CTR17 in the Non-FCM Sample
18	0.89	0.62
42	1.29	1.35
43	1.00	1.18

## Data Availability

The datasets generated and analyzed during the current study are available from the corresponding author upon reasonable request.
